# Piperlongumine in combination with EGFR tyrosine kinase inhibitors for the treatment of lung cancer cells

**DOI:** 10.32604/or.2024.053972

**Published:** 2024-10-16

**Authors:** SHAIL RAKESH MODI, TERRICK ANDEY

**Affiliations:** Department of Pharmaceutical Sciences, Massachusetts College of Pharmacy and Health Sciences, Worcester, MA 01608, USA

**Keywords:** Piperlongumine (PPL), Non-small cell lung cancer (NSCLC), Tyrosine kinase inhibitors (TKI), Mutation, Resistance

## Abstract

**Objectives:**

EGFR tyrosine kinase inhibitor (EGFR-TKI) therapies such as erlotinib and gefitinib are approved for the treatment of non-small cell lung cancer (NSCLC). However, the high incidence of acquired resistance to these EGFR-TKIs may preclude their effectiveness. Piperlongumine (PPL), an extract from the long pepper fruit (*Piper longum*), has been shown to possess anticancer properties. The purpose of the study was to investigate piperlongumine as an anticancer agent and to study a combination treatment approach with EGFR-TKIs against lung cancer cells.

**Methods:**

Anticancer efficacy of PPL, erlotinib (ERL), gefitinib (GEF), and cisplatin (CIS) were investigated in H1299 and H1975 cell lines. Cells were treated with PPL, ERL, GEF, and CIS alone, and in combination, cell viability was determined after 72 h. The mechanism of PPL-induced cytotoxicity was investigated via reactive oxygen species (ROS) induction, and apoptosis induction using acridine orange/ethidium bromide staining and flow cytometry. The effect of treatment on EGFR-mediated oncogenic signaling was investigated by immunoblotting for mitogenic and apoptotic markers.

**Results:**

PPL exhibited a potent cytotoxic effect in H1299 and H1975 cells compared to ERL, GEF, and CIS. Combination treatments of PPL with GEF and ERL showed significant reductions in cancer cells compared to control in both cell lines, which were associated with apoptotic induction, but without significant ROS induction. Compared to control, PPL with GEF significantly increased apoptotic cell death in H1975as confirmed with flow cytometry. Treatment with PPL alone and in combination induced anti-mitogenic and apoptotic responses at the molecular level.

**Conclusion:**

PPL sensitized lung cancer cells to EGFR-TKI and induced potent cytotoxic effects at low concentrations.

## Introduction

Lung cancer is one of the leading causes of mortality worldwide with estimated deaths of approximately 125 thousand in the United States in 2024 [1]. One of the major lung cancer biomarkers is the epidermal growth factor receptor (EGFR) [2]. EGFR is a tyrosine kinase receptor present on the cell surface, which when activated leads to various downstream signaling, resulting in cell growth, cell proliferation, and cell survival. EGFR-tyrosine kinase inhibitors (EGFR-TKIs) such as erlotinib and gefitinib target tumors with overexpression of EGFR. These tyrosine kinase inhibitors bind reversibly to the adenosine triphosphate (ATP) binding site of the EGFR receptor, which blocks its activation and downstream signaling [3,4]. These therapies are approved as first-line treatment for metastatic non-small cell lung cancer (NSCLC) and are effective against cells harboring certain EGFR mutations, the so-called activating mutations such as L858R mutation. However, treatment of NSCLC patients with first- and second-generation EGFR-TKIs has been associated with acquired resistance due to the emergence of T790M-EGFR mutation. EGFR-T790M is a secondary point mutation on exon 20 that replaces methionine for threonine at the amino acid position 790 on the EGFR tyrosine kinase [5] and is present in more than half of NSCLC patients [6]. Consequently, the affinity for the ATP increases and activates the downstream oncogenic and mitogenic signaling by the receptor [3,4]. On the other hand, activating mutations such as L858R–resulting from the deletion of leucine in exon 19 and the single amino acid substitution of arginine at the 858 amino acid position in exon 21–sensitize tumors to EGFR-TKI therapies [7]. Furthermore, the proto-oncogene MET (mesenchymal-epithelial transition, c-MET) plays an important role in encoding receptor tyrosine kinases. To address these challenges and improve the efficacy of the EGFR-TKIs, we investigated the role of Piperlongumine as a potential adjuvant therapy with EGFR-TKIs.

Piperlongumine is an amide alkaloid that is derived from the long pepper species, *Piper longum* L. Piperlongumine possesses various biological activities that include antiplatelet [8], antidepressant [9], neuroprotective [10], anti-inflammatory [11], antimicrobial [12] and anticancer properties [13]. Piperlongumine has demonstrated anticancer activity in various types of cancer such as colorectal cancer [14], prostate cancer [15], cervical cancer [16], intestinal cancer [17], lung cancer [18], and breast cancer [19]. Piperlongumine elicits cytotoxic effects by inducing apoptosis either by downregulating the anti-apoptotic protein Bcl2 or by activation of caspase-3 [20]. Additionally, piperlongumine also acts by generating reactive oxygen species (ROS) by disrupting redox homeostasis [21].

The current study aims to investigate the anti-cancer activity of piperlongumine as a monotherapy and in combination with EGFR-TKIs or cisplatin against NSCLC. The study also aims to highlight the molecular mechanism underlying the enhanced efficacy of the combination therapy approach. Cisplatin was included in the regimen as a comparator due to its wide-ranging cytotoxic and genotoxic activities across a broad spectrum of cancers, including NSCLC.

## Materials and Methods

### Reagents and cell lines

RPMI-1640 high glucose, fetal bovine serum (FBS), 1 × phosphate-buffered saline (PBS), 100 × antibiotic-antimycotic solution (penicillin (10–12 mg/mL)/streptomycin (10,000–12,000 U/mL)/amphotericin (25–30 μg/mL), fungin (500 µL), and trypsin-EDTA solution (0.25% Trypsin and 0.02% EDTA) were purchased from Thermo Fisher Scientific (Waltham, MA, USA). Piperlongumine (PPL), Gefitinib (GEF), Erlotinib (ERL), and Cisplatin (CIS) were purchased from (AK Scientific, Union City, CA, USA). H1299 (#CRL-5803) and H1975 (#CRL-5908) non-small cell lung cancer (NSCLC) cell lines were purchased from ATCC (Manassas, VA, USA). Both epithelial cell lines were selected due to their mutagenic difference; H1975 harbors the inherent L858R/T790M EGFR mutation while H1299 is an EGFR wild-type cell line. All the cell lines were grown in RPMI cell culture media supplemented with 10% FBS, 1× penicillin/streptomycin/amphotericin, and fungin, and maintained at 37°C in a humidified incubator (Thermo Fischer Scientific, Waltham, MA, USA) containing 5% CO_2_. All reagents used were of analytical or molecular grade.

### Cell proliferation

H1299 and H1975 cells were cultured in 182-cc flasks and allowed to reach 70–80% confluency. Cultured cells were treated with trypsin and seeded in a 96-well plate at a density of 1 × 10^5^ cells/mL and incubated overnight. Stock solutions of 0.1 M PPL, GEF, ERL, and CIS were prepared in dimethylsulfoxide (DMSO). The cells were treated with different concentrations of PPL, GEF, ERL, and CIS (i.e., 0.01, 0.1, 1, 10, and 100 μM) against their respective DMSO controls and incubated for 72 h. The media was removed, and the cells were washed with 100 μL of 1 × PBS. A volume of 100 μL resazurin dye (Alamar Blue, Milipore-Sigma, Burlington, MA, USA) (0.01 mg/mL) was added to each well and incubated for 2 h. Fluorescence was then measured at excitation/emission wavelengths of 530/590 nm, respectively, using a Synergy H1 plate reader (Agilent, Santa Clara, CA, USA). Experiments were performed in triplicates with at least three plates per treatment group. Results were plotted as line graphs of percentage cell viability against concentration. The drug concentration that resulted in 50% maximal cytotoxic effect (IC_50_) was computed using GraphPad Prism (version 8.0) and presented as average IC_50_ with SEM. CIS was included in the treatment regimen—as a comparator for the activities under investigation in the two NSCLC cell lines–as a standard chemotherapy agent due to its broad genotoxic and cytotoxic effect in different types of cancers, as well as limitation in NSCLC treatment as a result of innate and acquired resistance.

### Combination treatment

Combination treatment of PPL with GEF, ERL, or CIS was performed in H1299 and H1975 cells using fixed sub-IC_50_ concentrations of PPL, with varying concentrations of the other, and vice versa. Firstly, the concentration of PPL was fixed at 2.5 µM and the concentration of GEF, ERL, and CIS varied from 0.01 to 100 µM. Similarly, in H1299 cells and H1975 cells, respectively, the concentration of GEF (100 and 25 µM), ERL (65 and 25 µM), and CIS (25 and 50 µM) were fixed, and the concentration of PPL varied from 0.01 µM to 100 µM. Secondly, time-dependent cytotoxic effects of (concurrent *vs*. sequential) combination treatments were investigated at fixed concentrations of PPL with varying concentrations of GEF, ERL, and CIS at 0, 1, and 2 h, and vice versa. Although concentrations up to 1 µM of GEF and ERL are typically used for these types of studies, the authors explored a concentration range up to 100 µM to demonstrate the full spectrum of cytotoxic activity of these drugs, whether specific (at lower concentrations) or non-specific (at a high concentration of 100 µM). The data was analyzed as per the Chou-Talalay method by using the CompuSyn software (version 1.0), and the results were presented as combination index (CI) values, where CI < 1, =1, and >1 indicate synergism, additive effect, and antagonism, respectively [22].

### Reactive oxygen species (ROS) assay

H1299 and H1975 cells (5 × 10^4^ cells per well) were cultured in a 96-well black plate format and incubated overnight under standard culture conditions. Using the DCFDA/H2DCFDA Cellular ROS Assay Kit (Abcam, Waltham, MA, USA), wash buffer, DCFDA dye mixture (20 μM), and TBHP (100 μM) solution were prepared per the manufacturer’s protocol. Cell medium was removed from each well and the cells were washed with the wash buffer provided with the kit. This was followed by adding 100 μL of DCFDA dye mixture and subsequent incubation of the plate covered in aluminum foil for 45 min in an incubator. The DCFDA dye was then removed, and the cells were washed with the wash buffer, followed by the exposure of the cells to the respective single and combination treatments of PPL (2.5 μM), GEF (100, 25 μM), ERL (65, 25 μM), and CIS (25, 50 μM) in H1299 cells and H1975 cells, respectively; DMSO and TBHP (100 μM) were used as negative and positive controls, respectively. Fluorescence measurements were taken at excitation/emission wavelengths of 530/590 nm, respectively, using the Synergy H1 plate reader (Agilent, Santa Clara, CA, USA).

### Acridine orange/ethidium bromide (AO/EB) staining

H1299 and H1975 cells were seeded at a density of 5 × 10^4^ cells per ml in a 6-well plate format, and incubated overnight under standard cell culture conditions in RPMI-1640 medium without phenol red (Thermo Fischer Scientific, Waltham, MA, USA). The cells were exposed to single treatments of PPL (2.5 μM), GEF (100 μM), ERL (65 μM), CIS (25 μM), concurrent combinations (i.e., immediate treatment) of PPL + GEF, PPL + ERL, and PPL + CIS for 24 h. Similarly, sequential combination treatments (i.e., delayed treatment) for 24 h, with PPL (2.5 μM) followed by GEF (100 μM), ERL (65 μM), and CIS (25 μM) after 1 or 2 h, and vice versa were also performed. A working solution of acridine orange and ethidium bromide consisting of 10 μL of ethidium bromide solution (10 mg/mL) and 990 μL of acridine orange solution (0.1 mg/mL in 0.5 M acetate buffer) was prepared. An amount of 64 μL of the staining solution was added to each well and incubated for 10 min at 37°C. Fluorescence micrographs of the cells were acquired under green and red-light filters using the EVOS M7000 cell imaging system (Invitrogen, Waltham, MA, USA). Live cells will fluoresce a green color, whereas early apoptotic cells will produce a green to yellow color, and late apoptotic cells will stain red. Necrotic cells fluoresce orange color with a nuclear morphology resembling live cells.

### Flow cytometry

H1299 and H1975 cells (1 × 10^6^ cells) were cultured in 25-cc flasks and incubated overnight under standard culture conditions. The cells were treated with DMSO (control), PPL (2.5 μM), GEF (100 μM), ERL (65 μM), CIS (25 μM), and combinations of PPL with GEF; PPL with ERL; and PPL with CIS for 24 h. Cells were treated with trypsin, pelleted by centrifugation, and washed with 1 × PBS; 100 μL of the cell suspension in 1 × PBS was collected in the microcentrifuge tubes. A volume of 100 μL of Muse^TM^ Annexin V and Dead Cell Reagent (Cytex Biosciences, Fremont, CA, USA), previously equilibrated to room temperature was added into each cell suspension and remixed by pipetting 3–5 times. All tubes were kept incubated in the dark for 20 min at room temperature to ensure optimum staining. The mixture was then loaded into Muse^TM^ Cell Analyzer (Cytex Biosciences, Fremont, CA, USA) for measurement. Results were obtained as scatter plots with percent relative abundance of Annexin V-positive cells showing live, early apoptotic, late apoptotic, and dead cells.

### Western blot analysis

H1299 and H1975 cells were cultured in 182-cc flasks and treated with DMSO (control), PPL (2.5 μM), GEF (100 μM), ERL (65 μM), CIS (25 μM), and combinations of PPL with GEF; PPL with ERL; and PPL with CIS for 24 h. Using a cell scraper, the cells were collected in a test tube and centrifuged. Whole-cell protein lysates were prepared using RIPA Lysis Buffer [62.5 mM Tris-HCl (pH 6.8), 2% SDS, and 10% glycerol] with protease inhibitor according to manufacturer’s protocol (Thermo Fischer Scientific, Waltham, MA, USA). Protein concentrations were measured using Pierce^TM^ Bicinchoninic Acid Protein Assay Kit (Thermo Fischer Scientific, Waltham, MA, USA) according to the manufacturer’s protocol, and 12.5 µg/lane of total protein was loaded onto an AnyKD Mini-PROTEAN TGX Precast Protein Gel (Bio-Rad, Hercules, CA, USA) and separated by SDS-PAGE. Subsequently, proteins were transferred to 0.2 μM nitrocellulose membranes (Bio-Rad Laboratories, Hercules, CA, USA), and the membranes were blocked with 3% BSA solution in phosphate-buffered saline with 0.1% Tween-20 (PBST) overnight whilst shaking. The membranes were washed with PBST solution for 5 min three times, and then incubated with primary antibodies (1:1000) against EGF receptor (#4267, Cell Signaling), p-EGF receptor (#2234, Cell Signaling), EGF mutant (L858R mutant specific) receptor (#3197, Cell Signaling), Cleaved Caspase 3 (#9664, Cell Signaling), Cleaved Caspase 7 (#9491, Cell Signaling), and GAPDH (#2118, Cell Signaling), diluted in blocking solution at room temperature for 4 h. The membranes were then washed for 5 min three times and incubated with anti-rabbit IgG secondary antibody dilutions (1:2000) at room temperature for 2 h. The membranes were then exposed to a working solution of SuperSignal West Pico PLUS Chemiluminescent Substrate reagent (Thermo Fisher Scientific, Waltham, MA, USA) according to the manufacturer’s protocol, and the protein bands were visualized using ImageQuant^TM^ LAS 4000 (GE Healthcare, Chicago, IL, USA).

### Statistical analysis

All experiments were repeated at least 3 times and the differences were analyzed using One-way ANOVA followed by *post-hoc* Dunnett’s test. Statistical significance was determined at *p* < 0.05.

## Results

### Piperlongumine decreases cell proliferation in lung cancer cells

**Figure 1:**
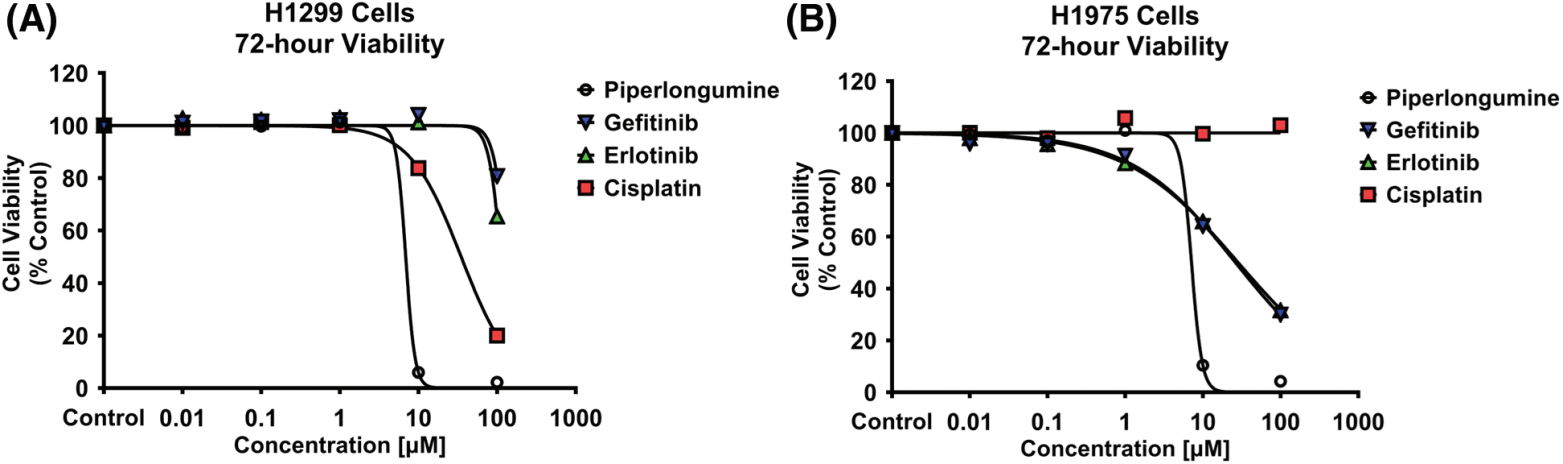
Alamar blue cell viability assay was performed in (A) H1299 and (B) H1975 for 72 h following individual treatments with PPL, GEF, ERL, and CIS at 0.01, 0.1, 1, 10, and 100 μM. Dimethyl sulfoxide (DMSO) was used as a control. Treatment with PPL resulted in a decrease in cell viability with an increase in concentration in both cell lines demonstrating a potential anticancer agent.

H1299 and H1975 cell viability was inhibited by PPL in a dose-dependent manner (Fig. 1A,B). The IC_50_ values were calculated for all the treatments, with the order of potency in H1299 and H1975 following PPL [5.84 ± 0.04 µM] > CIS [57.63 ± 3.06 µM] > ERL [138.88 ± 9.62 µM] > GEF [219.43 ± 22.69 µM], and PPL [6.05 ± 0.31 µM] > GEF [47.22 ± 3.97 µM] > ERL [51.92 ± 2.34 µM] > CIS [100 ± 3.06 µM], respectively. PPL exerted comparable potencies between the two cell lines, whereas GEF and ERL had superior cytotoxic effects against the H1975 cells *vs*. H1299 cells.

### Piperlongumine reverses EGFR-TKIs resistance in lung cancer cells

**Figure 2:**
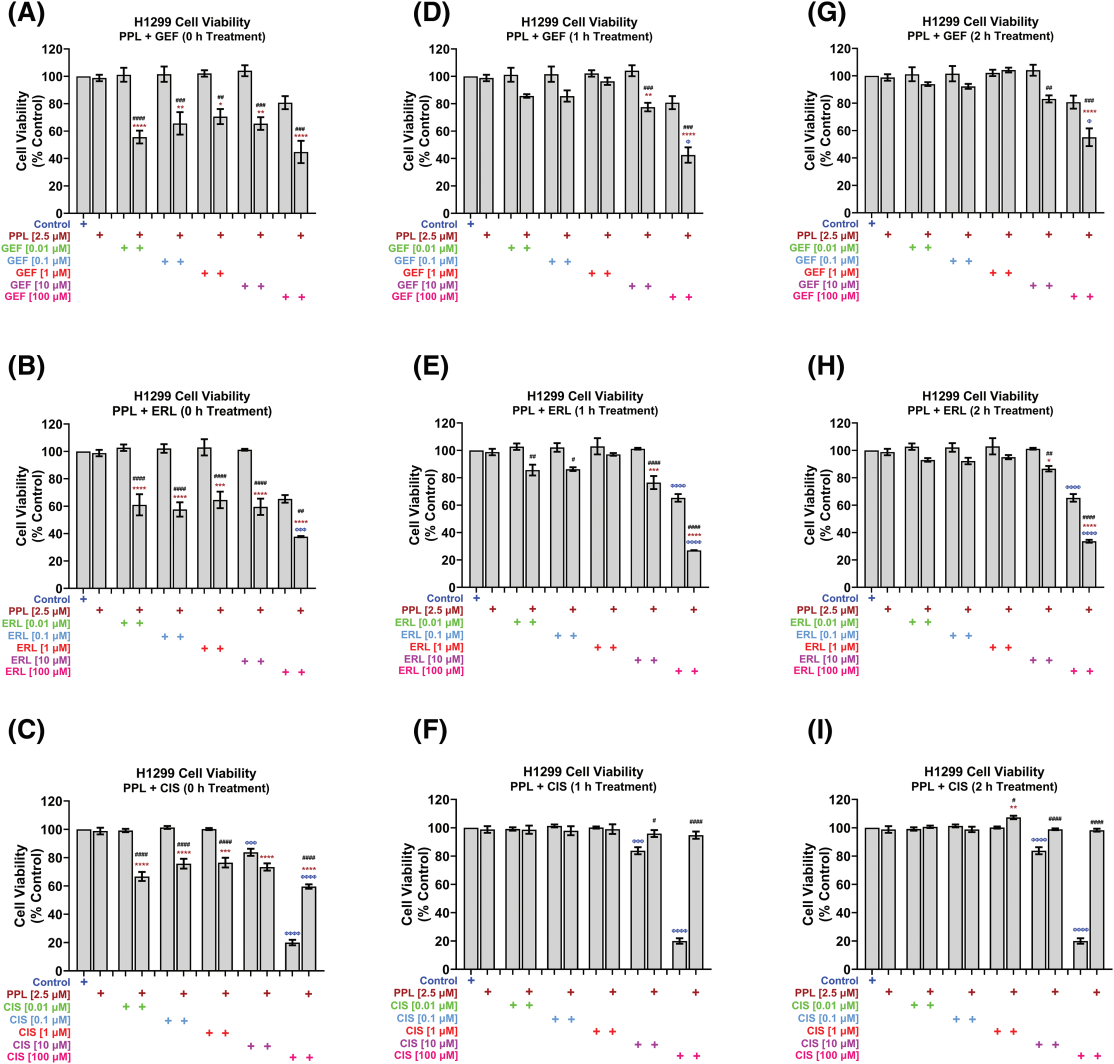
H1299 cell viability using alamar blue assay following combination treatments of PPL (2.5 µM) with varying concentrations of (A, D, G) GEF, (B, E, H) ERL, and (C, F, I) CIS. Combination treatment scheduling was either concurrent/immediate (0 h) or sequential/delayed (1 and 2 h). DMSO was used as a control. The concurrent treatment of EGFR-TKIs and PPL has significantly decreased cell viability at lower concentrations of EGFR-TKIs while the delayed treatment has decreased cell viability only at higher concentrations of EGFR-TKIs in the wild-type cells. The combination of PPL and CIS has decreased cell viability, however, the monotherapy of cisplatin has a better effect compared to the combination. Statistical analysis: One-way ANOVA with Sidak’s post-test. Compared to the control group: ^Φ^*p* < 0.05, ^ΦΦΦ^*p* < 0.001, and ^ΦΦΦΦ^*p* < 0.0001. Compared with the PPL (2.5 μM): *p < 0.05, **p < 0.01, ***p < 0.001, and ****p < 0.0001. Compared to the corresponding single treatment group of GEF, ERL, or CIS: ^#^*p* < 0.05, ^##^*p* < 0.01, ^###^*p* < 0.001 and ^####^*p* < 0.0001.

Concurrent/immediate combination treatment (i.e., 0 h treatment) with PPL at a fixed sub-IC_50_ concentration of 2.5 µM, with varying concentrations (0.01, 0.1, 1, 10, and 100 µM) of GEF, ERL, and CIS, resulted in significant decreases in H1299 cell viability at all concentrations of GEF [55.65 ± 4.69, 65.64 ± 8.22, 70.61 ± 5.48, 65.53 ± 4.62, and 44.78 ± 8.16 µM, respectively] (Fig. 2A), and ERL [61.04 ± 7.72, 57.60 ± 5.23, 64.57 ± 6.04, 59.54 ± 5.97, and 37.76 ± 0.46 µM, respectively] (Fig. 2B), compared to control and single treatments. Significant decreases in H1299 cell viability were also observed with PPL and CIS combination treatments up to 10 µM [66.69 ± 3.19, 75.72 ± 3.45, 76.48 ± 3.42, and 73.38 ± 2.57 µM, respectively], but not with 100 µM CIS, where combination treatment with PPL reversed the effect of CIS alone [59.59 ± 1.57 *vs*. 20.00 ± 1.95 µM, respectively] (Fig. 2C). The enhanced inhibitory effect that was observed with the concurrent co-treatment with PPL and GEF was only elicited at relatively higher concentrations of 10 and 100 µM GEF when co-treatments were delayed/separated by 1 h [77.52 ± 3.13 and 42.51 ± 5.62 µM] (Fig. 2D,G) and 2 h [83.13 ± 6.47 and 55.11 ± 6.47 µM] (Fig. 2E,H). The antagonistic effect of PPL on 100 µM CIS was also observed in the delayed/sequential treatments at 1 (Fig. 2F) and 2 h (Fig. 2I).

**Figure 3:**
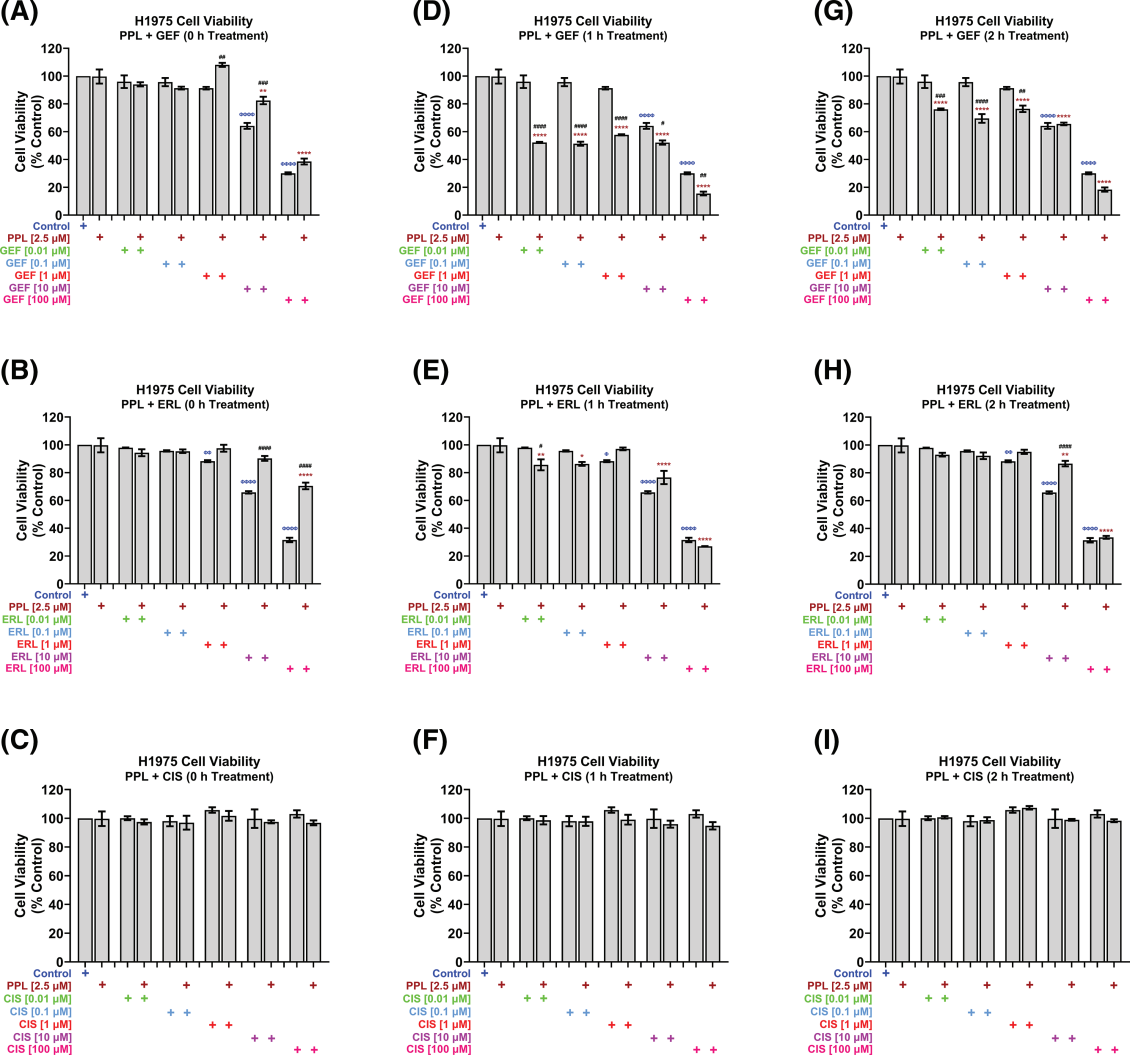
H1975 cell viability alamar blue assay following combination treatment of PPL (2.5 µM) with varying concentrations of (A, D, G) GEF, (B, E, H) ERL, and (C, F, I) CIS. Combination treatment scheduling was either concurrent/immediate (0 h) or sequential/delayed (1 and 2 h). DMSO was used as a control. The monotherapy of EGFR-TKIs had a significant decrease in cell viability compared to the concurrent combination of EGFR-TKIs and PPL. Delaying the addition of the EGFR-TKIs resulted in a significant decrease in cell viability in the mutant lung cancer cells. The combination of PPL and CIS had no significant changes in the cell viability against H1975 cells. Statistical analysis: One-way ANOVA with Sidak’s post-test. Compared to the control group: ^Φ^*p* < 0.05, ^ΦΦ^*p* < 0.01, ^ΦΦΦΦ^*p* < 0.0001. Compared with the PPL (2.5 μM): *p < 0.05, **p < 0.01, and ****p < 0.0001. Compared to the corresponding single treatment group of GEF, ERL, or CIS: ^#^*p* < 0.05, ^##^*p* < 0.01, ^###^*p* < 0.001 and ^####^*p* < 0.0001.

In H1975 cells, concurrent treatment of 2.5 µM PPL with GEF (Fig. 3A) and ERL (Fig. 3B) at concentrations up to 1 µM had no effect on cell viability, while antagonizing the effects of GEF and ERL at 10 and 100 µM. The concurrent combination of PPL and CIS did not significantly affect H1975 cell viability across all concentrations (Fig. 3C). However, when co-treatments were delayed by 1 h (Fig. 3D) and 2 h (Fig. 3G), significant decreases in cell viability were observed across all combination groups compared to control, and single treatments of PPL and GEF. One-hour delayed co-treatments of PPL with ERL significantly decreased cell viability at 0.01 and 0.1 µM (Fig. 3E). However, PPL antagonized ERL effects at 1 and 10 µM, with no effect on ERL at 100 µM; similar results were observed with 2 h delayed treatments of PPL and ERL (Fig. 3H). The combination of PPL and CIS did not significantly affect H1975 cell viability across all concentrations for either concurrent or delayed treatments (Fig. 3F,I).

### Piperlongumine and EGFR-TKI co-treatments inhibit mitogenic potentials of H1299 and H1975 cells

**Figure 4:**
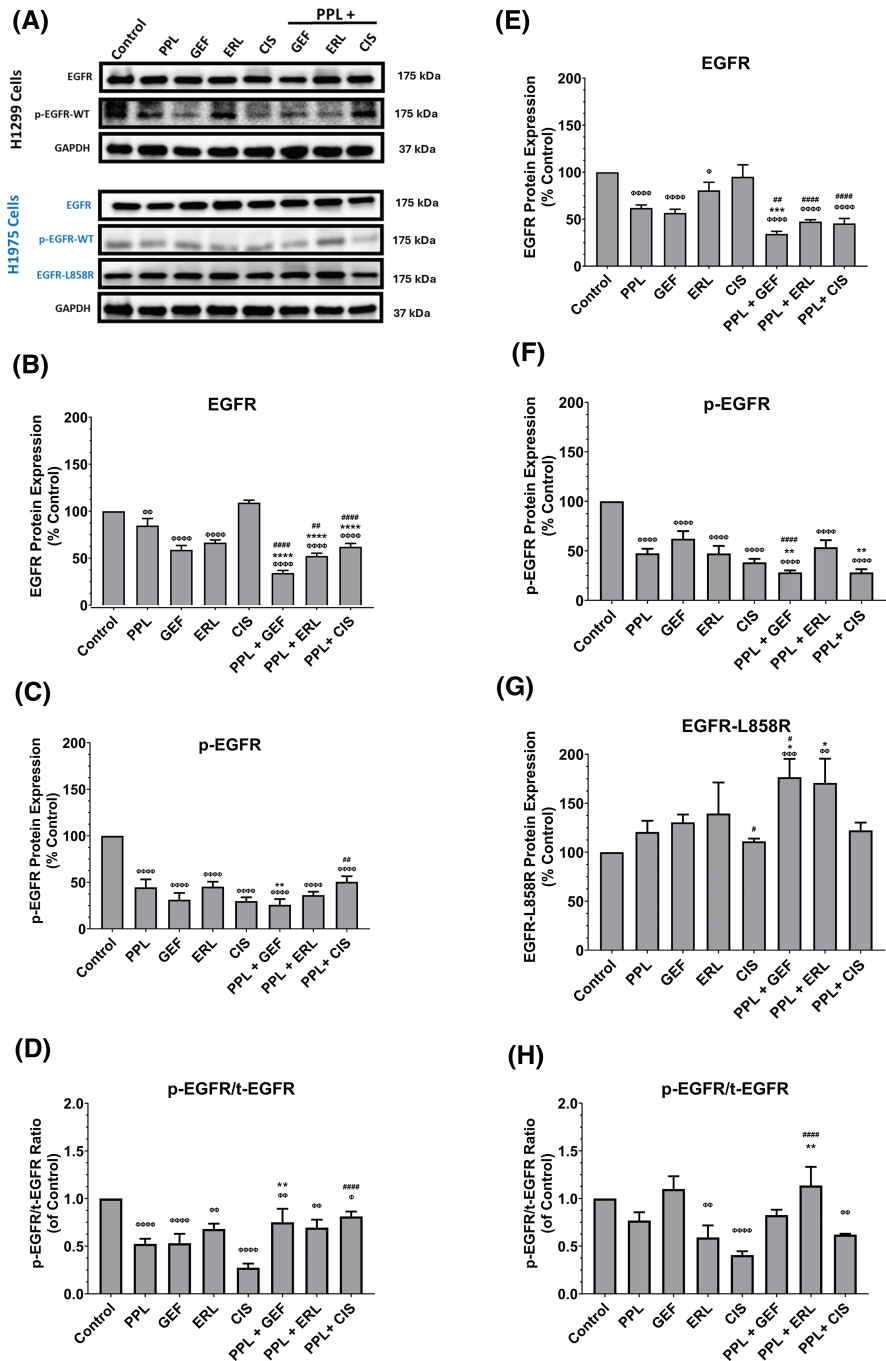
(A) Representative western blots of EGFR, p-EGFR, and EGFR-L858R (mutant) proteins in H1299 and H1975 cells following treatments with single and combination regimens of PPL, GEF, ERL, and CIS; DMSO was used as control. GAPDH was used as a loading control. (B–D) Graphs showing the relative expression of EGFR, p-EGFR, and p-EGFR/t-EGFR proteins in H1299 cells. (E–H) Graphs showing the relative expression of EGFR, p-EGFR, and EGFR-L858R in H1975 cells. Statistical analysis: One-way ANOVA with Sidak’s post-test. Compared to the control group: ^Φ^*p* < 0.05, ^ΦΦ^*p* < 0.01, ^ΦΦΦ^*p* < 0.001, and ^ΦΦΦΦ^*p* < 0.0001. Compared with the PPL (2.5 μM): *p < 0.05, **p < 0.01, ***p < 0.001, and ****p < 0.0001. Compared to the corresponding single treatment group of GEF, ERL, or CIS: ^#^*p* < 0.05, ^##^*p* < 0.01, ^####^*p* < 0.0001.

The representative blots of EGFR (wild-type and L858R mutant), and p-EGFR protein expression are depicted in Fig. 4A. The combination treatments of PPL with GEF, ERL, or CIS in H1299 cells, resulted in significant reductions in EGFR (wild-type) and p-EGFR protein expression, compared to control and individual treatments (Fig. 4B,C respectively). In H1299 cells, combination treatment of PPL with GEF resulted in a significant decrease in EGFR (34%), compared to control (100%) and individual treatments of PPL and GEF (85% and 59%, respectively) (Fig. 4B); p-EGFR expression was also decreased in the combination group (26%) *vs.* individual treatments of PPL and GEF (45% and 31%, respectively) (Fig. 4C). Similarly, the combination of PPL with ERL exhibited significant decreases in EGFR (52%) *vs.* individual treatments of PPL (85%) and ERL (67%) (Fig. 4B), whereas p-EGFR level in the PPL and ERL combination was also decreased (36%) compared to PPL (45%) and ERL (45%) individually (Fig. 4C). The combination of PPL and CIS, compared to individual treatments, afforded a reduction of EGFR, but not p-EGFR (Fig. 4B,C respectively). The relative ratios of p-EGFR to total EGFR (p-EGFR/t-EGFR) showed downregulation of EGFR phosphorylation (p-EGFR) relative to control, in H1299 cells with a combination treatment of PPL and GEF (0.75-fold), PPL and ERL (0.69-fold), and PPL and CIS (0.81-fold) (Fig. 4D). However, the effects observed in the combination treatments were lower compared to the corresponding individual treatments.

In H1975 cells, combination treatments of PPL and GEF resulted in significant decreases in EGFR (wild-type) (34%), compared to control (100%) and individual treatments (62% and 57%, respectively) (Fig. 4E); p-EGFR expression was also decreased in the combination group (28%) *vs.* individual treatments of PPL and GEF (48% and 62%, respectively) (Fig. 4F). Similarly, the combination of PPL with ERL exhibited significant decreases in EGFR (47%) *vs.* individual treatments of PPL (62%) and ERL (81%) (Fig. 4E), whereas p-EGFR level in the PPL and ERL combination was decreased (54%) compared to PPL (48%) and ERL (47%) individually (Fig. 4F). Expression of mutated EGFR (L858R mutant) showed increased expression in all treatment groups across all treatment groups compared to control, with the increases being significant in the PPL-GEF (177%) and PPL-ERL (171%), relative to control (100%) and individual treatments of PPL (121%), GEF (131%) and ERL (139) (Fig. 4G). The relative ratio of p-EGFR to total EGFR showed significant decreases in the ERL and CIS individual treatments, and the converse in the combination groups, with significant upregulation seen in the PPL and ERL group (113%) (Fig. 4H), compared to the control.

### Piperlongumine induces a synergistic response with EGFR-TKIs

**Figure 5:**
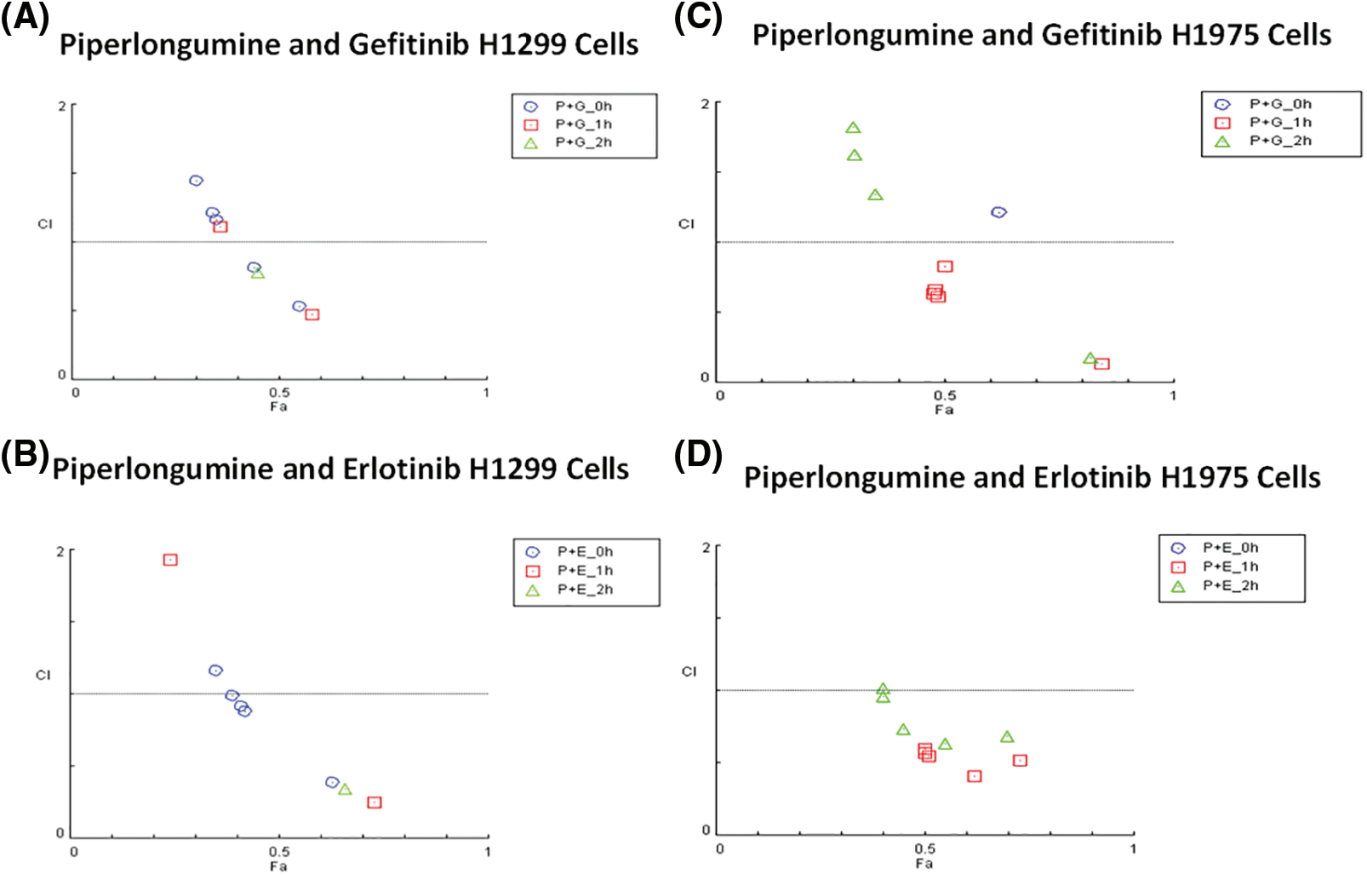
Combination index plots characterizing the interactions occurring with combination treatment of PPL with GEF, or ERL in (A and B) H1299 and (C and D) H1975 via CompuSyn. Combination Index, CI (*y*-axis) values over 1-antagonistic effect, CI less than 1–synergistic effect, and CI equal to 1–additive effect. Fraction associated, Fa (*x*-axis) represents the effect of the drug. Each panel shows the effect of combination treatment schedules based on either concurrent/immediate (0 h) or sequential/delayed (1; 2 h) administration with PPL and GEF (P+G_0 h; P+G_1 h; P+G_2 h, respectively), or PPL and ERL (P+E_0 h; P+E_1 h; P+E_2 h, respectively).

Synergistic effects were observed for 100 µM of GEF, ERL, and CIS with 2.5 µM PPL in H1299 cells (Fig. 5A,B). In H1299, however, synergistic effects were only observed at the lowest concentration of 0.01 µM GEF with 2.5 µM PPL (CI = 0.81) following concurrent treatment (0 h), with other combination treatments of GEF with PPL showing additive to antagonistic effects across all time points. Similarly, synergism was only observed with concurrent treatments of PPL with 0.1 µM ERL (CI = 0.88) but not with other concentrations or time points. In H1975, synergistic effects were elicited with 1 h delayed treatments for all combinations of 2.5 µM PPL with GEF (CI = 0.13–0.83) (Fig. 5C) and ERL (CI = 0.41–0.59) (Fig. 5D). Additional CI data for all treatment combinations are contained in Table S1A–D.

### A combination of PPL and EGFR TKI increases the ROS levels

**Figure 6:**
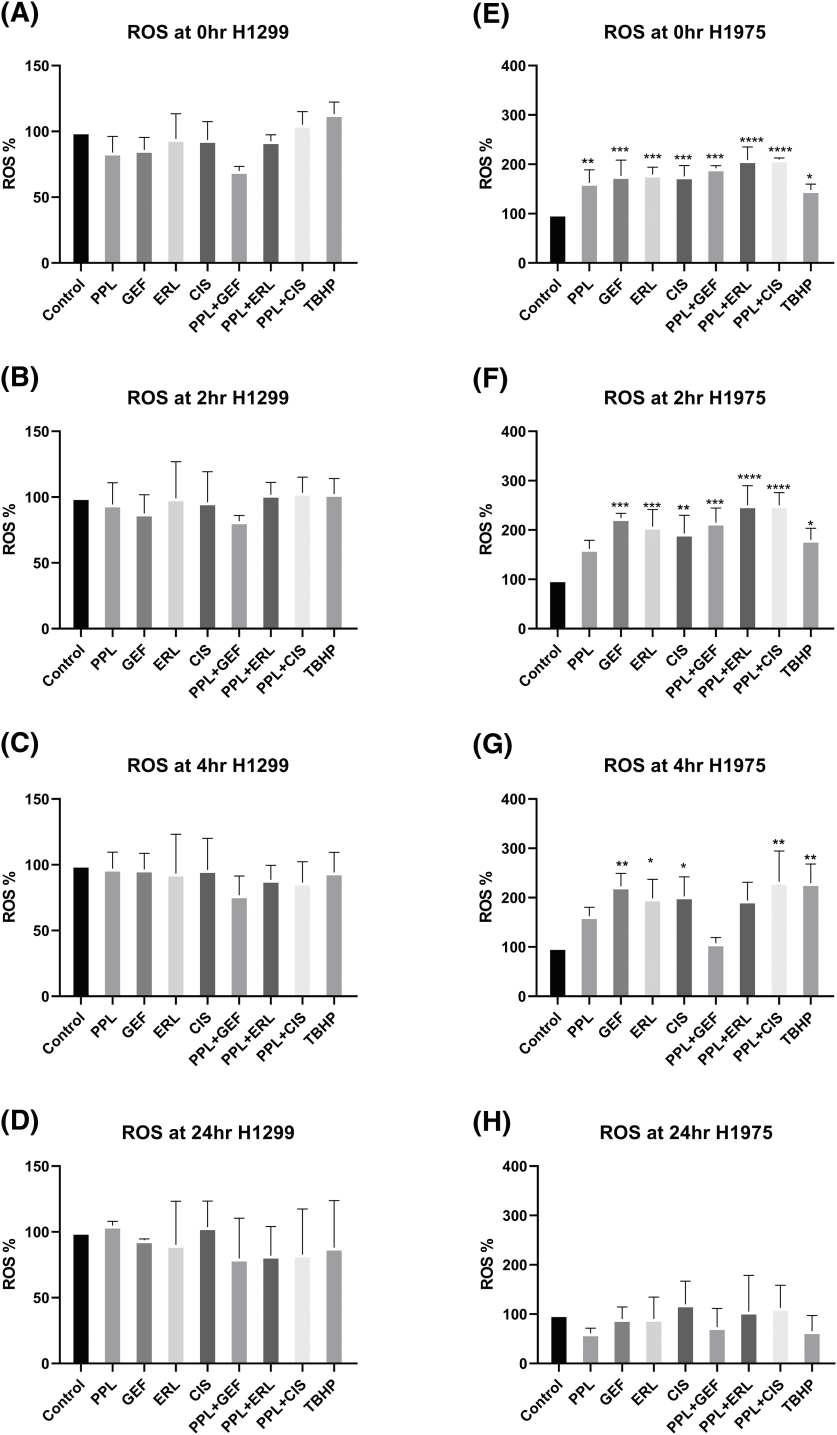
Results of ROS assay in (A–D) H1299 and (E–H) H1975 cells at 0, 2, 4, and 24 h following concurrent/immediate (0 h) single and combination treatments with PPL (2.5 μM), GEF (100 and 25 μM), ERL (65 and 25 μM), and CIS (25 and 50 μM) in H1299 cells and H1975 cells, respectively; DMSO and TBHP (100 μM) were used as untreated and positive control, respectively. One-way ANOVA with Dunnet’s post-test. Compared to the control group: *p < 0.05, **p < 0.01, ***p < 0.001 and ****p < 0.0001.

In H1299 cells, no significant increase in ROS levels was observed with either single or combination treatments (Fig. 6A–D). In H1975 cells, however, significantly elevated ROS levels were observed in individual as well as combination treatment groups, compared to control (Fig. 6E–H). The elevated ROS levels were sustained and peaked at 2 h (Fig. 6E,F) and dissipated from 4 to 24 h (Fig. 6G,H, respectively). The combination of PPL with GEF produced 191.80 ± 0.52% of ROS levels at 0 h which was increased to 215.03 ± 0.46% at 2 h. Similarly, the combination of PPL with ERL produced 208.90 ± 0.48% of ROS levels at 0 h which was increased to 250.20 ± 0.46% at 2 h. The combination treatment produced greater ROS levels than the individual treatment of PPL, GEF, and ERL (Fig. 6). These results suggest that PPL induces ROS and in addition to EGFR-TKIs, the levels increase. Moreover, a selective mechanism was observed between the two cell lines, which requires further investigation.

### Piperlongumine and EGFR-TKI induce apoptosis in lung cancer cells

**Figure 7:**
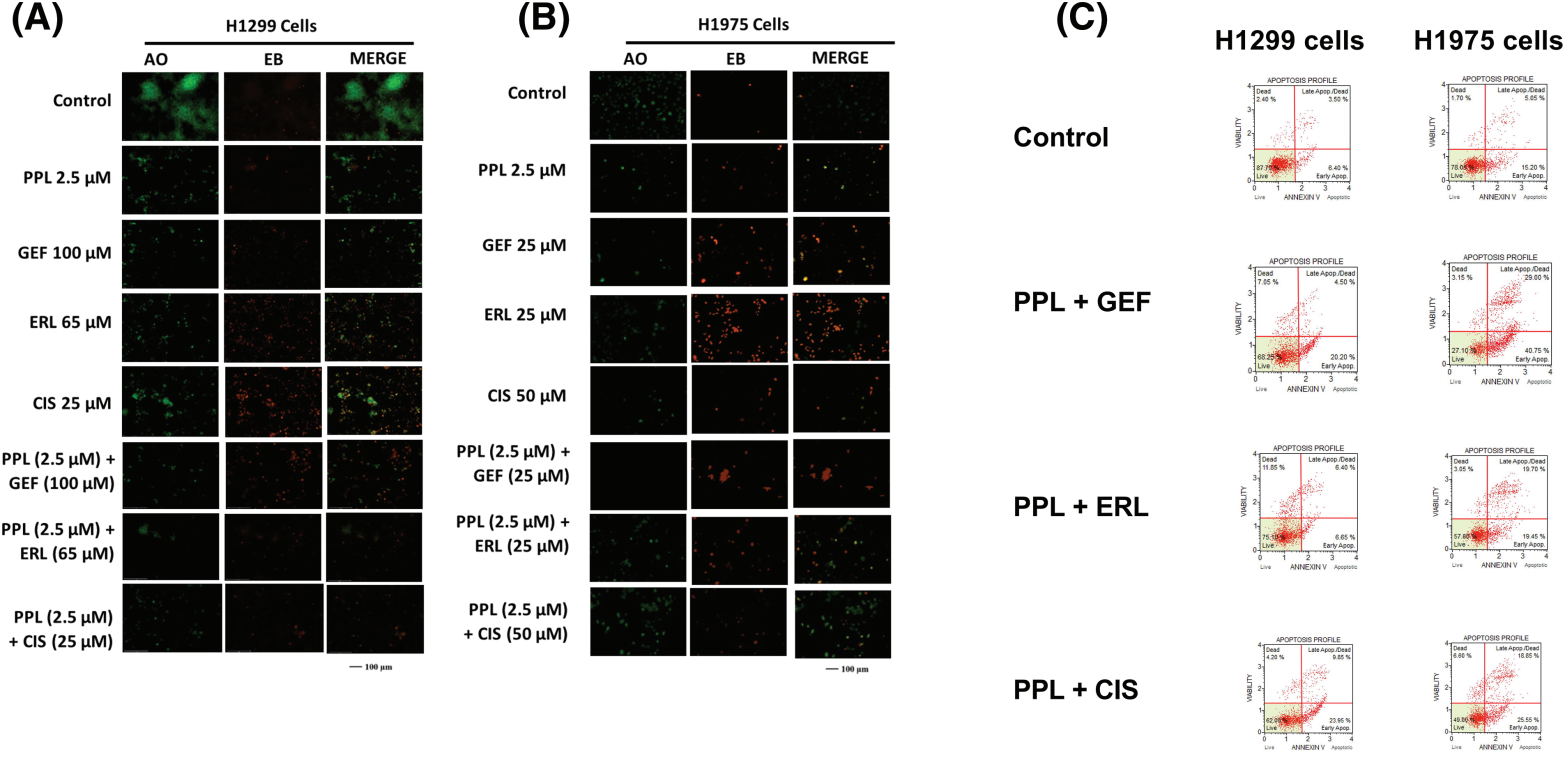
Fluorescence micrographs showing results of Acridine orange/Ethidium Bromide (AO/EB) staining following individual treatments with Control (DMSO), PPL, GEF, ERL, and CIS and their combination in (A) H1299 cells and (B) H1975 cells. The green color denotes live cells, and the orange-to-red color indicates different stages of apoptosis from early to late apoptosis. (C) Representative flow cytometry scatter plots showing the distribution of live, early apoptotic, late apoptotic, and dead cells in H1299 cells (left panel) and H1975 cells (right panel) following treatment with Control (DMSO), PPL + GEF, PPL + ERL, and PPL + CIS. For H1299 cells the concentrations used were PPL (2.5 µM), GEF (100 µM), ERL (65 µM), CIS (25 µM), PPL + GEF (2.5 + 100 µM), PPL + ERL (2.5 + 65 µM), and PPL + CIS (2.5 + 25 µM). For H1975 cells the concentrations used were PPL (2.5 µM), GEF (25 µM), ERL (25 µM), CIS (50 µM), PPL + GEF (2.5 + 25 µM), PPL + ERL (2.5 + 25 µM), and PPL + CIS (2.5 + 50 µM).

Qualitative assessment of induction of apoptosis following single and combination treatment of PPL and EGFR-TKIs was performed using Acridine Orange/Ethidium Bromide (AO/EB) staining. Acridine orange (AO) is a nucleic acid staining dye that binds to live and dead cells and fluoresces green color. Ethidium bromide (EB) is a fluorescent dye that binds to dead cells and fluoresces red color. Treatment with PPL individually, as well as in combination with GEF and ERL resulted in a change of the morphology of the cells consistent with apoptotic induction (i.e., shrunken cytoplasm, cell nucleus fragmentation, apoptotic bodies, or chromatin condensation) (Fig. 7A–C). Delayed treatment produced a relatively greater response compared to immediate treatment (Fig. S1A–D).

### Piperlongumine and EGFR-TKI combination increase apoptotic cells in lung cancer cells

Quantitative assessment of apoptosis induction using flow cytometric analysis showed increased cell death with combination treatment in both cell lines. Compared to control groups in H1299 (13.79%) and H1975 (25.90%), the combination treatment of PPL with GEF produced 21.54% and 59% apoptotic cells, respectively, whereas PPL with ERL produced 16.29% and 36.45% apoptotic cells, respectively (Fig. 7C). Additional results of apoptotic induction from individual treatments are shown in Figs. S2 and S3. At the molecular level, combination treatment produced a significant increase in protein expression of cleaved caspase 3 in H1299 and H1975 cells. The combination of PPL and GEF increased the protein levels in both cell lines by an average of 70%, while the combination of PPL and ERL increased the protein levels by approximately 30%, compared to the control (Fig. S4A,C). While a significant increase in protein levels of cleaved caspase 7 occurred in H1299 cells, the reverse was observed in H1975 cells (Fig. S4B,D).

## Discussion

EGFR mutations are caused by multiple factors that result in malignant transformations. An increase in the EGFR tyrosine kinase activity leads to the activation of various downstream signaling which results in cell growth, cell proliferation, and cell survival activities [23]. Molecularly targeted therapies, such as EGFR-TKIs are designed to exploit the differences in the biology of cancer and non-cancer cells, thereby allowing for the specific therapeutic targeting of such tumors. However, over a period as short as 7 months, tumors may acquire resistance against many of these therapies. Combinatorial treatment strategies where two or more therapies with different mechanistic and therapeutic properties are used to synergize their anticancer potencies have demonstrated success [17,24,25]. The current study demonstrates the success of a combinatorial therapeutic approach with PPL and EGFR-TKIs (GEF and ERL) to overcome resistance and enhance the anticancer effect of lung cancer cells.

In the present study, PPL decreased the cell viability of NSCLC cells (H1299 and H1975) at non-toxic concentrations (sub-IC_50_ 2.5 µM), which was 3-fold lower than the 15 µM dose of PPL that was shown to be tolerated in a toxicity study conducted *in vitro* and *in vivo* in non-cancerous cells [26]. PPL exhibited greater anticancer potency in both H1975 and H1299 cells compared to the EGFR-TKIs-GEF and ERL, both of which showed relatively nominal and variable potencies between the two cell lines. However, GEF and ERL were more effective against H1975 compared to H1299. The variable responsiveness could be due to the mutagenic differences of EGFR between the two cell lines; H1975 cells harbor inherent compound mutations of (L858R and T790M) that may enable sensitivity (L858R) or resistance (T790M) to GEF and/or ERL, compared to H1299 cells with heterozygous/wild-type EGFR expression [7]. It is worth noting that GEF and ERL were designed to specifically target mutated EGFR, which may explain the differences in their efficacies. Additionally, genotypic differences in the L858R and T790M mutation status of EGFR in the same type of cell line may result in different responsiveness to GEF and ERL [27]. For example, studies have shown that the double inherent mutation L858R/T790M predisposes cancer cells to be significantly more resistant to GEF and ERL compared to a single mutation of either L858R or T790M [28]. Further, NSCLC cells have been shown to carry other types of mutations that conspire with EGFR mutation to enhance the resistance phenotype. For example, MET amplification in lung cancer cells has been associated with an increase in the resistance potential of H1975 to ERL, possibly due to the crosstalk that has been demonstrated to exist between the two receptors [29]. While the current study did not investigate MET expression in the two cell lines, previous studies have implicated H1975, but not H1299 with the constitutive amplification of MET [30]. Further, H1299 is constitutively characterized with a null-p53 and KRAS mutated status that has been shown to amplify its resistance potential to EGFR-TKIs such as GEF and ERL [31].

To overcome the resistance threshold posed by H1299 (to a greater extent) and H1975 and highlight the potential differences in responsiveness to first-generation EGFR-TKIs due to the EGFR mutation status, a combinatorial approach with PPL and GEF or ERL was undertaken to exploit the mechanistic differences between the two classes of therapies to potentiate the anticancer effect. The study also highlights the temporal modulation of co-administration of a fixed concentration of PPL (2.5 µM) with varying concentrations of GEF or ERL (i.e., concurrent/immediate; 0 h *vs*. sequential/delayed; 1 and 2 h) to understand the potential effects of physical or chemical interactions between the two therapies, and how these may inform the manner of their administration to elicit optimal therapeutic outcomes. However, the results of this study show that H1299 cells and H1975 cells, while varying in degrees, demonstrated resistance to CIS with concentrations as high as 100 µM, in some instances. This is consistent with observations in published studies that have provided mechanistic evidence of innate and acquired NSCLC to CIS [32,33].

Consideration must be given to the utility of either concurrent or sequential co-administration of therapies in the treatment of cancer as either approach could significantly impact the course and outcome of treatment, as well as the overall patient health and safety. Various *in vitro*, *in vivo*, and clinical trial studies have demonstrated the value of one scheduling approach over the other, and in some cases, showing no significant variations in outcomes from either [34]. In this study, cell-specific responses to the combination treatment of PPL with either GEF, ERL, or CIS were observed. In H1299, concurrent/immediate co-administration of the treatments (0 h) was consistent in decreasing cell viability for all combination regimens, with inhibitory effects observed at concentrations of GEF, ERL, and CIS as low as 0.01 µM. However, sequential/delayed treatments (1 and 2 h) did not afford greater potency, with significant inhibitory effects only seen at combinations of PPL with the maximum concentrations of GEF and ERL (100 µM). The relative superiority observed with the concurrent treatments in H1299 is consistent with results observed from *in vitro* and *in vivo* studies in lung cancer [35], and prostate and breast cancer [36]. The influence of concurrent treatment in H1975 cells, however, was quite unremarkable across all treatment regimens; and only nominally significant inhibitions were observed with sequential treatment schedules of 1 h with EGFR-TKI concentrations of 0.01, 0.1 and 10 µM. Notably, the combination treatments in H1975 appeared to favor PPL, by enhancing its inhibitory effects from non-therapeutic levels observed at the sub-IC_50_ concentration of 2.5 µM. Future studies will focus on the impact of a combination treatment with a focus on H1975, with PPL concentration ranging from 2.5 to 5 µM to further explore its adjuvant therapeutic potency. These results were consistent with combination index (CI) values showing synergistic interactions occurring at these combined concentrations and treatment schedules.

Mechanistically, the enhanced inhibitory effects were associated with decreases in the basal expression and activation of EGFR in both cell lines. Combination treatment of PPL with GEF and ERL resulted in increased expression of EGFR-L858R protein levels in H1975 cells, which is consistent with an enhanced TKI effect resulting from an upregulated status of the therapeutically favorable activating mutation of EGFR-L858R. Piperlongumine has been shown to mediate cytotoxic responses in lung cancer via ROS- and apoptosis-dependent pathways [20]. Cell-specific and time-dependent regulation of ROS production by PPL, GEF, ERL, and CIS individually, and in combination was observed, with increases in ROS induction occurring in H1975 up to 2 h, but not in H1299. Notably, ROS production was relatively higher in single treatments of GEF, ERL, and CIS compared to PPL, with additive effects resulting from combination treatments. These observations are consistent with studies showing the induction of ROS by PPL [37], GEF [38], ERL [39], and CIS [40] in NSCLC and other cancer types. The cytotoxic effects resulting from combination treatments of PPL with GEF, ERL, and CIS manifested in phenotypic programmed-cell death responses which were validated both qualitatively (AO/EB) and quantitatively (flow cytometry) and were associated with upregulation of cleaved caspase 3 in H1299 cells, but less so of cleaved caspase 7, which was only overexpressed in the PPL-GEF combination group (Fig S5). A similar expression pattern of cleaved caspase 3 was observed in H1975, however, accompanied by a downregulation of cleaved caspase 7. The role of caspases 3, 7, and 9 in mediating apoptosis via ROS-dependent and independent pathways has been established. Notably, activation/cleavage of caspase 3 by caspase 9 has been associated with the inhibition of ROS production, while cleaved caspase-7 has been demonstrated to function in facilitating the detachment of apoptotic cells, but not ROS production [41]. These dynamics may provide a rational basis for the observation in Fig. 5, where neither individual nor combination treatments resulted in ROS production in H1299. The expression of cleaved caspase 3 in H1975 is very nominal relative to H1299, which might explain the relatively high ROS production, perhaps due to limited inhibitory influences by cleaved caspase 3. Future studies will explore the full spectrum of caspase-dependent intrinsic and extrinsic apoptotic pathways to better understand the mechanism underlying the phenotypic observations.

The current study is limited to the data provided by two NSCLC cell lines with different phenotypes, a wild-type, and an inherent mutated cell line. The inclusion of additional mutated cell lines would have provided a more robust result. Also, PPL desensitizing EGR-TKI’s can be explored more by including different cell lines. Additionally, the study did not investigate the cell-specific differences in ROS production as a mechanism for inducing cell death by PPL in combination with EGFR-TKIs in the two cell lines. Moreover, the study claims would be strengthened with *in vivo* assessment which the authors plan as future studies investigating the combination treatments in a mice model of NSCLC.

## Conclusion

GEF and ERL are first-generation EGFR-TKIs whose therapeutic utility in NSCLC with EGFR harboring the exon 19 deletions or exon 21 (L858R) substitutions is beset by increasing rates of acquired resistance, resulting in shorter progression-free survival (PFS) periods, with median values ranging between 9 to 13 months [42]. The current study leverages a combinatorial treatment approach using PPL–an alkaloid compound from the long pepper plant–with GEF and ERL to induce cytotoxic responses in two NSCLC cell types with wild-type and mutated EGFR. Significantly, the study highlights the dynamic mechanisms that mediated the strong cytotoxic response elicited by the combination regimen in a cell-specific, time-dependent, and pathway-specific manner. Importantly, the strong cytotoxic responses obtained with a minimal and non-toxic dosing schedule of PPL individually and in combination, provide a strong empirical basis for further investigations into its application as adjunctive therapy in NSCLC.

## Supplementary Materials

Figure S1 Acridine orange/Ethidium bromide (AO/EB) staining was performed to assess the induction of apoptotic cells following the time-delayed combination treatments in H1299 (A and B) and H1975 cells (C and D). In Figures A and C, PPL is added first followed by the other treatment. In Figures B and D, GEF, ERL, and CIS are added first followed by PPL. The AO panel represents live cells (green fluorescence). The EB panel represents the apoptotic/dead cells (red fluorescence) and the Merge panel represents live and dead cells.

Figure S2 Representative flow cytometry graphs generated using MuseTM Cell Analyzer following PPL, GEF, ERL and CIS treatment in H1299 (above) and H1975 (Below). For H1299 cells the concentrations used were PPL (2.5 µM), GEF (100 µM), ERL (65 µM), CIS (25 µM). For H1975 cells the concentrations used were PPL (2.5 µM), GEF (25 µM), ERL (25 µM), CIS (50 µM). The graph depicts the percentage of Live cells, early apoptotic, late apoptotic, and dead cells in different quadrants.

Figure S3 Graphical representation of flow cytometric analysis of A) H1299 and B) H1975 cells. Dimethyl sulfoxide (DMSO) solution was used as a control. Compared with the control group, *p < 0.05, **p < 0.01, ***p < 0.001.

Figure S4 A) to D) Relative expression of proteins in H1299 and H1975 cells compared to control. Dimethyl sulfoxide (DMSO) solution was used as a control. Compared with the control group, *p < 0.05, **p < 0.01, ****p < 0.0001.

## Data Availability

All data generated or analyzed during this study are included in this published article (and its supplementary information files). Article and supplementary information will be provided upon consent of the authors.

## Supplementary Materials

The supplementary material is available online at https://doi.org/10.32604/or.2024.053972.
