# Supplementary figures and images for: Piperlongumine in combination with EGFR tyrosine kinase inhibitors for the treatment of lung cancer cells
Source: Oncol Res. 2024 Oct 16;32(11):1709–21. doi: 10.32604/or.2024.053972 (PMC11497197; doi:10.32604/or.2024.053972)

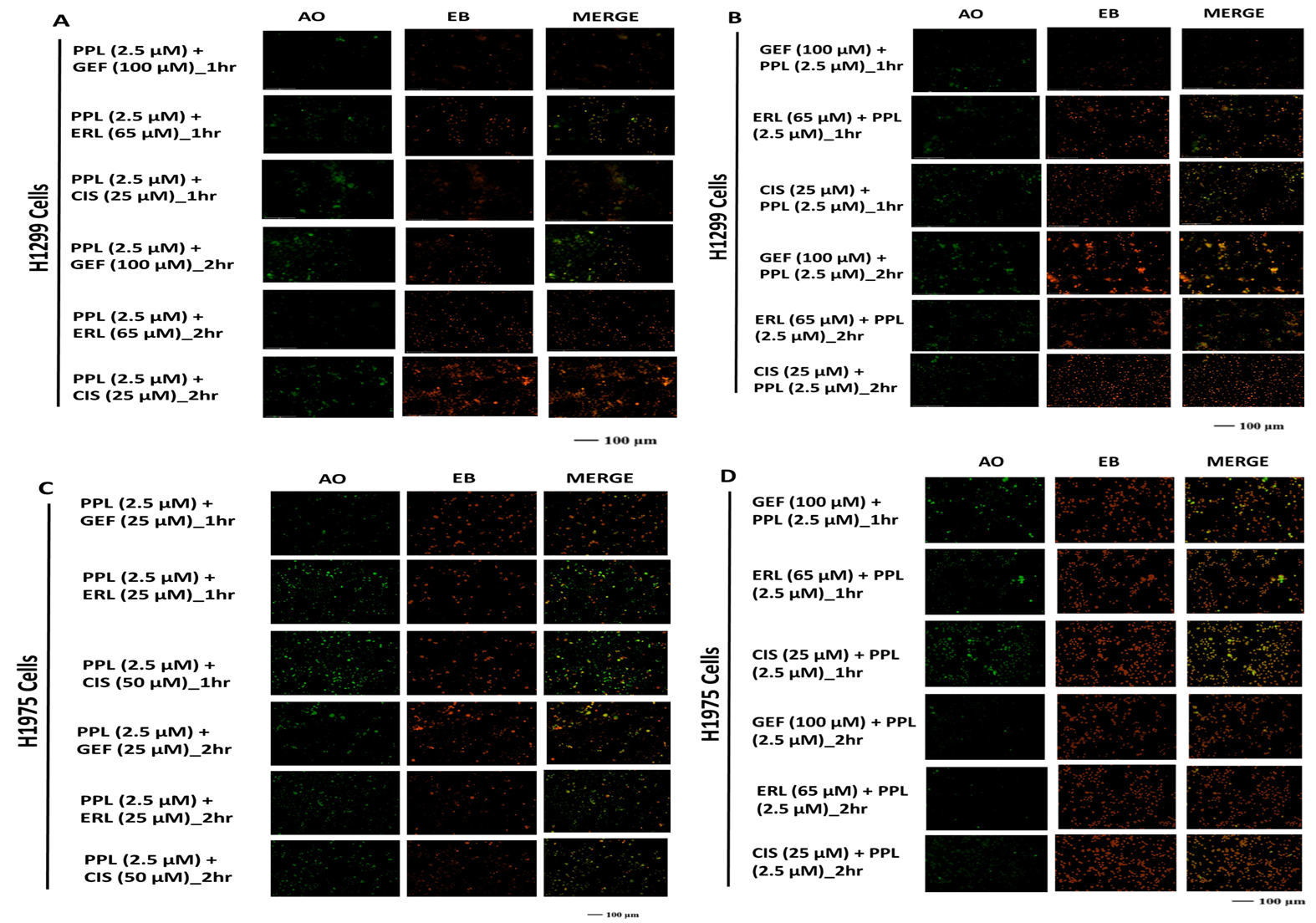

Supplement: Figure S1 [file OncolRes-32-53972-s001.tif]

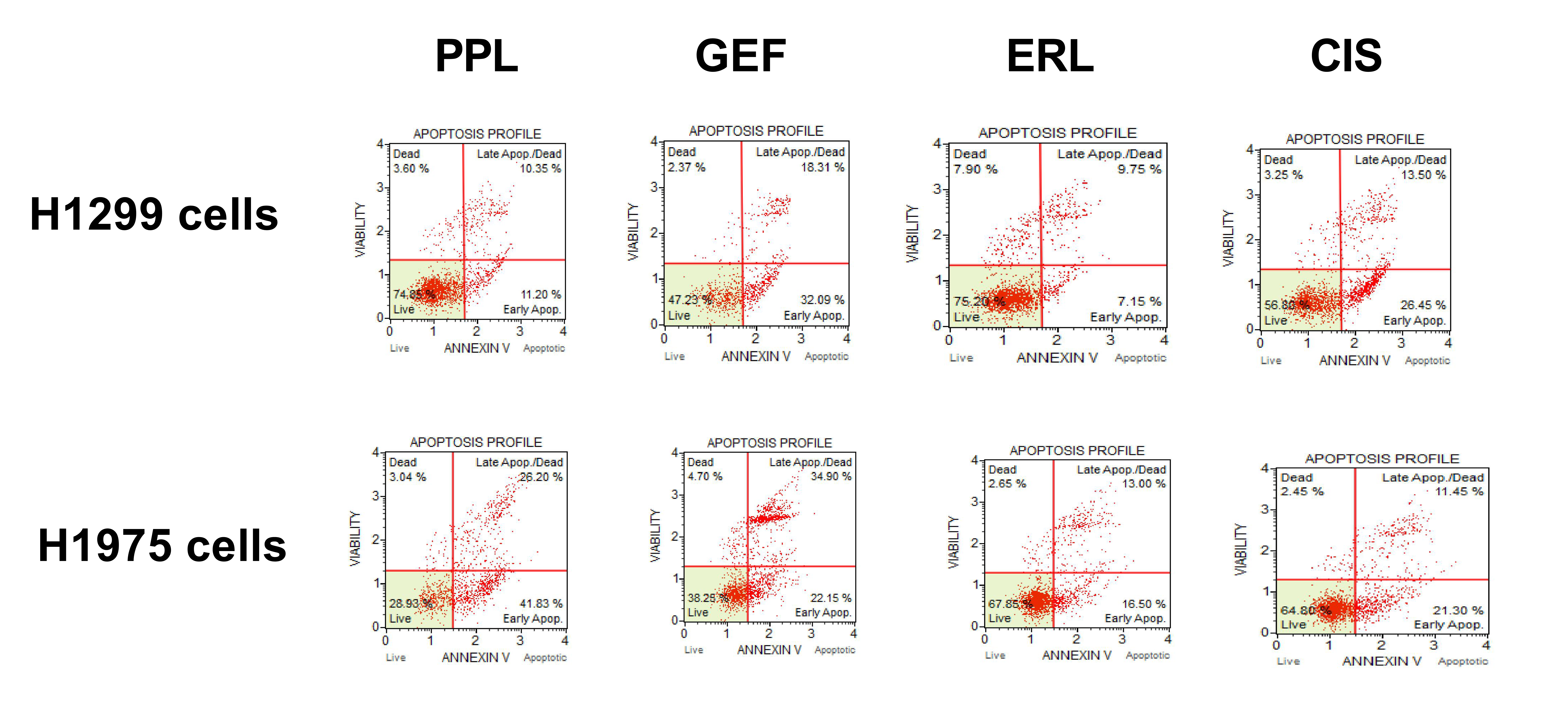

Supplement: Figure S2 [file OncolRes-32-53972-s002.tif]

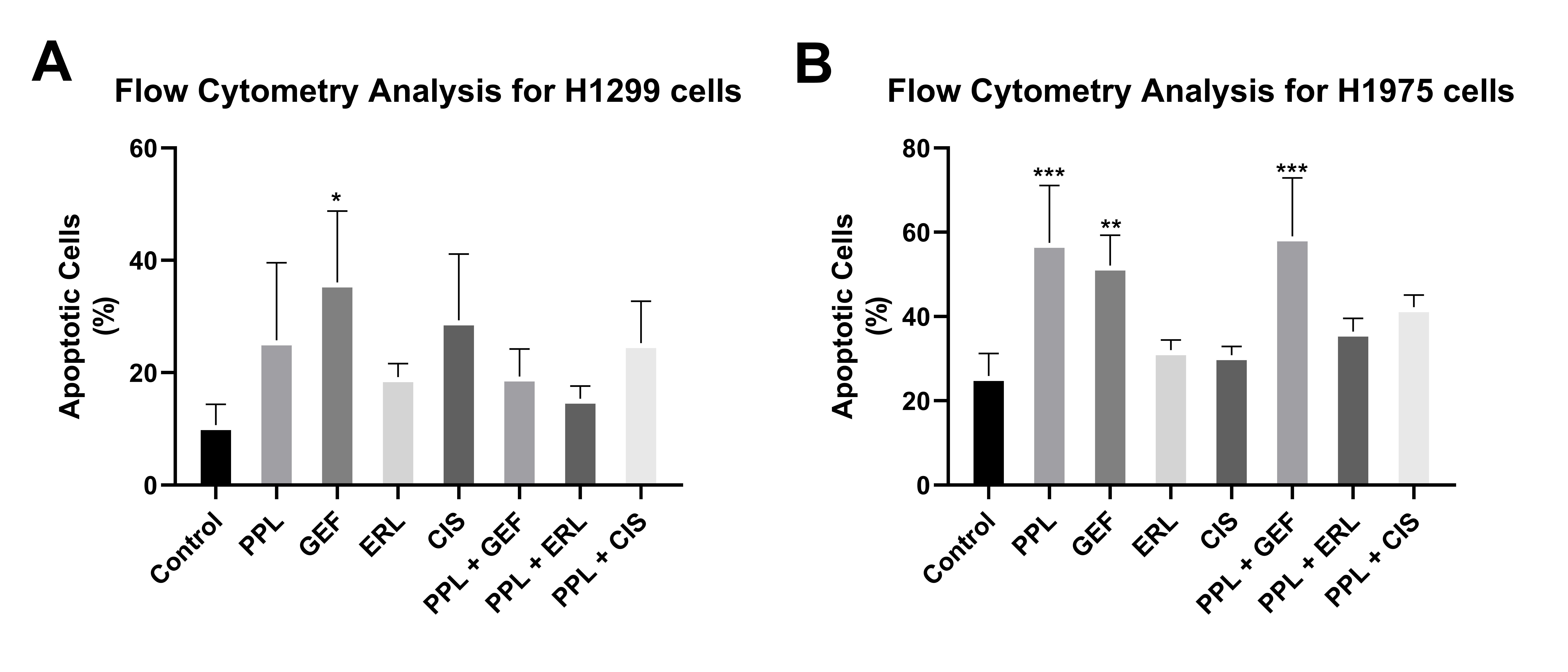

Supplement: Figure S3 [file OncolRes-32-53972-s003.tif]
