# Supplementary material for: Piperlongumine in combination with EGFR tyrosine kinase inhibitors for the treatment of lung cancer cells
Source: Oncol Res. 2024 Oct 16;32(11):1709–21. doi: 10.32604/or.2024.053972 (PMC11497197; doi:10.32604/or.2024.053972)
Supplement: Supplementary file 5 [file OncolRes-32-53972-s001.docx]

Table S1: Combination Index (CI) values for PPL and EGFR-TKIs in NSCLC generated via CompuSyn

1. Piperlongumine and Gefitinib Combination Index values in H1299 cells

|  | **Dose Piperlongumine (****μM)** | **Dose**  **Gefitinib (μM)** | **Effect** | **Combination Index** |
| --- | --- | --- | --- | --- |
| **Immediate Treatment 0 h** | 2.50 | 0.01 | 0.25 | 0.81 |
|  | 2.50 | 0.10 | 0.25 | 1.16 |
|  | 2.50 | 1.00 | 0.26 | 1.44 |
|  | 2.50 | 10.00 | 0.30 | 1.21 |
|  | 2.50 | 100.00 | 0.50 | 0.53 |
| **Delayed Treatment 1 h** | 2.50 | 0.01 | 0.14 | 3.49 |
|  | 2.50 | 0.10 | 0.14 | 3.49 |
|  | 2.50 | 1.00 | 0.10 | 5.17 |
|  | 2.50 | 10.00 | 0.23 | 2.03 |
|  | 2.50 | 100.00 | 0.59 | 0.47 |
| **Delayed Treatment 2 h** | 2.50 | 0.01 | 0.07 | 7.49 |
|  | 2.50 | 0.10 | 0.08 | 6.53 |
|  | 2.50 | 1.00 | 0.01 | 60.12 |
|  | 2.50 | 10.00 | 0.17 | 2.90 |
|  | 2.50 | 100.00 | 0.45 | 0.78 |

1. Piperlongumine and Erlotinib Combination Index values in H1299 cells

|  | **Dose Piperlongumine (μM)** | **Dose**  **Erlotinib (μM)** | **Effect** | **Combination Index** |
| --- | --- | --- | --- | --- |
| **Immediate Treatment 0 h** | 2.50 | 0.01 | 0.31 | 0.99 |
|  | 2.50 | 0.10 | 0.42 | 0.88 |
|  | 2.50 | 1.00 | 0.36 | 1.16 |
|  | 2.50 | 10.00 | 0.41 | 0.91 |
|  | 2.50 | 100.00 | 0.63 | 0.39 |
| **Delayed Treatment 1 h** | 2.50 | 0.01 | 0.14 | 3.52 |
|  | 2.50 | 0.10 | 0.14 | 3.61 |
|  | 2.50 | 1.00 | 0.10 | 5.19 |
|  | 2.50 | 10.00 | 0.24 | 1.93 |
|  | 2.50 | 100.00 | 0.73 | 0.25 |
| **Delayed Treatment 2 h** | 2.50 | 0.01 | 0.07 | 7.49 |
|  | 2.50 | 0.10 | 0.08 | 6.53 |
|  | 2.50 | 1.00 | 0.05 | 10.59 |
|  | 2.50 | 10.00 | 0.14 | 3.64 |
|  | 2.50 | 100.00 | 0.66 | 0.35 |

C) Piperlongumine and Gefitinib Combination Index values in H1975 cells

|  | **Dose Piperlongumine (μM)** | **Dose**  **Gefitinib (μM)** | **Effect** | **Combination Index** |
| --- | --- | --- | --- | --- |
| **Immediate Treatment 0 h** | 2.50 | 0.01 | 0.06 | 18.45 |
|  | 2.50 | 0.10 | 0.09 | 10.96 |
|  | 2.50 | 1.00 | 0.01 | 767.98 |
|  | 2.50 | 10.00 | 0.18 | 10.99 |
|  | 2.50 | 100.00 | 0.62 | 1.21 |
| **Delayed Treatment 1 h** | 2.50 | 0.01 | 0.48 | 0.64 |
|  | 2.50 | 0.10 | 0.49 | 0.61 |
|  | 2.50 | 1.00 | 0.48 | 0.66 |
|  | 2.50 | 10.00 | 0.50 | 0.83 |
|  | 2.50 | 100.00 | 0.85 | 0.13 |
| **Delayed Treatment 2 h** | 2.50 | 0.01 | 0.24 | 2.44 |
|  | 2.50 | 0.10 | 0.31 | 1.63 |
|  | 2.50 | 1.00 | 0.30 | 1.83 |
|  | 2.50 | 10.00 | 0.35 | 1.35 |
|  | 2.50 | 100.00 | 0.82 | 0.18 |

D) Piperlongumine and Erlotinib Combination Index values in H1975 cells

|  | **Dose Piperlongumine (μM)** | **Dose**  **Erlotinib (μM)** | **Effect** | **Combination Index** |
| --- | --- | --- | --- | --- |
| **Immediate Treatment 0 h** | 2.50 | 0.01 | 0.05 | 23.56 |
|  | 2.50 | 0.10 | 0.05 | 24.28 |
|  | 2.50 | 1.00 | 0.05 | 38.89 |
|  | 2.50 | 10.00 | 0.10 | 40.76 |
|  | 2.50 | 100.00 | 0.31 | 17.80 |
| **Delayed Treatment 1 h** | 2.50 | 0.01 | 0.50 | 0.57 |
|  | 2.50 | 0.10 | 0.51 | 0.54 |
|  | 2.50 | 1.00 | 0.50 | 0.59 |
|  | 2.50 | 10.00 | 0.62 | 0.41 |
|  | 2.50 | 100.00 | 0.73 | 0.52 |
| **Delayed Treatment 2 h** | 2.50 | 0.01 | 0.45 | 0.73 |
|  | 2.50 | 0.10 | 0.40 | 0.96 |
|  | 2.50 | 1.00 | 0.40 | 1.02 |
|  | 2.50 | 10.00 | 0.55 | 0.64 |
|  | 2.50 | 100.00 | 0.70 | 0.69 |
